# Enhanced activity of doxorubicin in drug resistant A549 tumor cells by encapsulation of P-glycoprotein inhibitor in PLGA-based nanovectors

**DOI:** 10.3892/ol.2013.1711

**Published:** 2013-11-27

**Authors:** LIANG XU, HUA LI, YUBIN WANG, FANG DONG, HUANGBING WANG, SHUTONG ZHANG

**Affiliations:** 1 Department of Critical Care Medicine, Wuhan City Hospital No. 3 and Tongren Hospital of Wuhan University, Wuhan, Hubei 430060, P.R. China; 2 Department of Gynaecology & Obstetrics, Tianyou Hospital Affiliated to Wuhan University of Science and Technology, Wuhan, Hubei 430064, P.R. China; 3 Medical College, Wuhan University of Science and Technology, Wuhan, Hubei 430065, P.R. China; 4 Department of Radiology, Wuhan Center and Second Hospital, Wuhan, Hubei 430014, P.R. China

**Keywords:** poly(D,L-lactide-*co*-glycolide) nanoparticles, drug resistance, efflux pump, P-glycoprotein

## Abstract

Effective chemotherapy remains an important issue in the treatment of drug resistant cancer. The aim of the present study was to establish novel polymeric nanoparticles composed of the antitumor drug, doxorubicin (DOX), and an inhibitor of the drug efflux pump-associated protein, P-glycoprotein (P-gp), in order to overcome drug resistance in tumor cells. Poly(D,L-lactide-*co*-glycolide) (PLGA), DOX-loaded PLGA (PLGA-DOX), P-gp inhibitor (cyclosporin A; CsA)-coated PLGA (PLGA-CsA) and DOX and CsA co-loaded PLGA (PLGA-DOX-CsA) nanoparticles were prepared using solvent evaporation. The size distribution, ζ potential and electron microscopy observations of the nanoparticles were characterized. Accumulation and efflux assays were performed using confocal and fluorescence-activated cell sorting (FACS), and the pump activity of P-gp was detected through FACS. The uptake of the nanoparticles and the viability of Taxol-resistant A549 cells treated with various nanoparticles were analyzed via FACS *in vitro.* Furthermore, the tumor growth and survival rates of A549-Taxol-bearing mice were monitored *in vivo*. Prepared particles were nanosized and the efflux rates of PLGA-DOX and PLGA-DOX-CsA were significantly decreased compared with the free DOX. Drug efflux pump activity was effectively inhibited by the PLGA-CsA and PLGA-DOX-CsA groups compared with the PLGA, PLGA-DOX and free DOX groups. Cell viability results demonstrated that PLGA-DOX and PLGA-DOX-CsA induced the increased death of A549-Taxol cells. *In vivo* tumor models demonstrated that PLGA-DOX and PLGA-DOX-CsA markedly inhibited the tumor growth and improved the survival rate of A549-Taxol-bearing mice. Antitumor drug and drug efflux pump inhibitor co-loaded nanoparticles offer advantages to overcome the drug resistance of tumors and highlight new therapeutic strategies to control drug resistant tumors.

## Introduction

Lung cancer is a major cause of cancer mortality worldwide. Resistance to antitumor drugs is a vital obstacle to overcome for tumor chemotherapy (1). In the past several decades, the following strategies have been developed to circumvent drug resistance: i) Inhibition of the functions of the multidrug resistance (MDR)-associated protein (MRP) or ATP-dependent membrane drug efflux pump protein, P-glycoprotein (P-gp), by several generation modulators to decrease the efflux rate of antitumor drugs; ii) blocking of molecular mechanisms via pathways to defeat and possibly prevent drug resistance. Villanueva *et al* revealed that a combination therapy involving a MAPK/ERK kinase (MEK) and v-raf murine sarcoma viral oncogene homolog B1 (BRAF) inhibitor may be an effective strategy to conquer drug resistance in melanoma (2); acquired resistance to BRAF inhibitors mediated by a RAF kinase switch in melanoma may be overcome by co-targeting MEK and IGF-1R/PI3K; and iii) increasing the uptake and accumulation of antitumor drugs using therapeutic nanoparticles or liposomes for drug delivery (3–5). To date, the drug resistance of tumors remains a big challenge.

As a polymeric nanoparticulate system, poly(D,L-lactide-*co*-glycolide) (PLGA) has gained attention for the preparation of a wide variety of delivery systems containing several drugs due to its biodegradable and biocompatible properties and low toxicity (6,7). The etiology of MDR may be multifactorial, but the classic resistance to cytotoxic drugs has often been associated with the overexpression of P-gp, a 170-kd ATP-dependent membrane transporter, which acts as a drug efflux pump (8–10). Previously, the inhibition of P-gp as a method of reversing MDR has been extensively studied (11–13). Numerous agents that modulate the function of P-gp have been identified, including calcium channel blockers (14), calmodulin antagonists (15), steroidal agents (16), protein kinase C inhibitors, immunosuppressive drugs, antibiotics and surfactants. Cyclosporin A (CsA) has been demonstrated as a broad-spectrum MDR modulator in a number of previous studies (17,18).

In the current study, to achieve an improved therapeutic effect of antitumor drug resistant cancer, a more optimized delivery system using PLGA was adopted, that not only increases drug [doxorubicin (DOX)] uptake, but also reduces the efflux rate of DOX by co-delivering the P-gp inhibitor, CsA.

## Materials and methods

### Cells and agents

The paclitaxel-resistant non-small cell lung cancer A549 cell line (A549-Taxol), cultured in DMEM medium (10% FBS, 1% penicillin and 1% streptomycin) and 0.25% trypsin solution, was purchased from Invitrogen Life Technologies (Carlsbad, CA, USA). PLGA, DOX and CsA were purchased from Sigma-Aldrich (St. Louis, MO, USA), phycoerythrin (PE)-labeled mouse anti-human P-gp antibody was purchased from eBioscience, Inc. (San Diego, CA, USA) and anti-P-gp antibody (265/F4) for western blot analysis was provided by Wuhan University (Wuhan, China).

### Flow cytometry

The expression levels of human P-gp on the surfaces of the A549-Taxol cells were examined by fluorescence-activated cell sorting (FACS) analysis using PE-labeled anti-human P-gp antibodies. The cells were incubated with or without the antibody for 45 min at 4°C, followed by washing twice with PBS. Fluorescence staining levels were measured using FACSCalibur (BD Biosciences, Franklin Lakes, NJ, USA).

### Western blotting

Cell lysates of A549-Taxol cells were separated by SDS-PAGE and then electrotransferred onto a polyvinylidene difluoride membrane. The membrane was incubated with 1 mg/ml anti-P-gp monoclonal antibody (265/F4) purchased from Abcam (Cambridge, MA, USA), followed by washing 3 times and treatment with peroxidase-conjugated goat anti-mouse secondary antibody (Amersham Pharmacia Biotech, Amersham, UK). Subsequently, the membrane-bound antibody was visualized with the Enhanced Chemiluminescence Plus Detection kit (Amersham Pharmacia Biotech).

### Preparation of nanovectors

PLGA, CsA-coated PLGA (PLGA-CsA), DOX-loaded PLGA (PLGA-DOX) and DOX and CsA-loaded PLGA (PLGA-DOX-CsA) were prepared using a modified procedure of oil in water single emulsion solvent evaporation. The organic phase consisted of PLGA polymer in a dichloromethane-acetone mixture (2:1) and the aqueous phase contained P-gp inhibitor, CsA and DOX (alone or together). The organic phase was emulsified with the aqueous phase using an Ultra-Turrax model T25 (IKA^®^-Werke GmbH & Co. KG, Staufen, Germany) at 14,000 rpm in an ice bath for 5 min. The organic mixture was then removed rapidly by evaporation under nitrogen gas at 37°C. The particles were centrifuged at 100,000 × g for 30 min, washed 3 times in distilled water and freeze-dried for use.

### Surface morphology, particle size and ζ potential analysis

The surface morphology of PLGA, PLGA-CsA, PLGA-DOX and PLGA-DOX-CsA was examined by a Hitachi model H-800 transmission electron microscope (TEM) (Hitachi, Ltd., Tokyo, Japan). Freshly prepared particles were washed with ddH_2_O at 100,000 × g for 1 h and then re-suspended in ddH_2_O for analysis. Samples were transferred to a cuvette for dynamic light scattering analysis to measure the size distribution or subjected to an electric field for ζ potential determination using Zetasizer Nano ZS (Malvern Instruments, Malvern, UK).

### Accumulation of free DOX, PLGA-DOX and PLGA-DOX-CsA

The A549-Taxol cells (2×10^4^) were seeded in 24-well tissue culture plates and incubated with free DOX, PLGA-DOX and PLGA-DOX-CsA nanoparticles for 6, 12, 24 and 48 h. A DOX concentration equivalent to 2 μg/ml was maintained in the solutions. Following incubation, the cells were washed 3 times to remove the DOX, PLGA-DOX or PLGA-DOX-CsA that had not been internalized. The cells were subsequently observed and images were captured using fluorescence microscopy (BX53; Olympus, Corp., Tokyo, Japan).

### In vitro cytotoxicity assay

The A549-Taxol cells (2×10^3^) were plated into 96-well plates and incubated with free PLGA (200 mg/ml), PLGA-CsA (1 mg/ml CsA), free DOX (10 mg/ml), PLGA-DOX (10 mg/ml DOX) and PLGA-DOX-CsA (10 mg/ml DOX and 1 mg/ml CsA ) for 6, 12, 24, 48 and 72 h. Next, 10 ml WST-1 reagents were added to each well and incubated at 37°C for 4 h. Finally, the absorbance was measured using a Perkin-Elmer 2030 VICTOR X Multilabel Plate Reader (Perkin-Elmer, Waltham, MA, USA) at 450 nm.

### Antitumor activity of PLGA-DOX and PLGA-DOX-CsA

The *in vivo* antitumor efficacy of PLGA-DOX and PLGA-DOX-CsA was assessed in female BALB/c background SCID mice (body weight, 18–20 g). The A549-Taxol cells (2×10^5^) were subcutaneously injected into the mice. The mice were randomly divided into six groups (PBS, PLGA, PLGA-CsA, free DOX, PLGA-DOX and PLGA-DOX-CsA), with six mice in each group. The mice were treated with a single intravenous (i.v.) injection of a 10 μg/ml dose, equivalent to DOX, in each group. The control group of mice received a single i.v. injection of PBS or free PLGA particles. At predetermined time intervals, the tumor volume was determined by measuring the tumor dimensions using digital calipers and then calculated according to the following formula: Tumor volume (mm^3^) = width × (length / 2)^2^. Survival rates of the mice were observed and calculated for 60 days.

### Statistical analysis

A one- and two-way analysis of variance and Student’s t-test were used to determine statistical significance. P<0.05 was considered to indicate a statistically significant difference.

## Results

### High expression of P-gp in A549-Taxol cells

**Figure 1:**
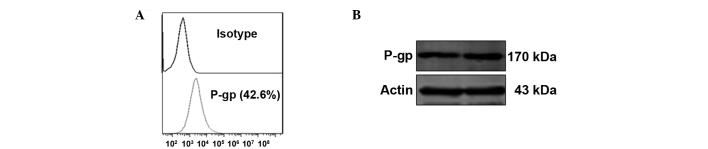
Expression of P-gp in A549-Taxol cells. P-gp expression in the Taxol-resistant A549 cells was analyzed by (A) FACS and (B) western blot analysis. P-gp, P-glycoprotein; FACS, fluorescence-activated cell sorting.

FACS and western blot analysis were performed to confirm the expression of P-gp in the A549-Taxol cells. As predicted, the cells were positive for P-gp in the FACS (Fig. 1A) and western blot analysis results (Fig. 1B).

### Characteristics of PLGA, PLGA-DOX, PLGA-CsA and PLGA-DOX-CsA

**Figure 2:**
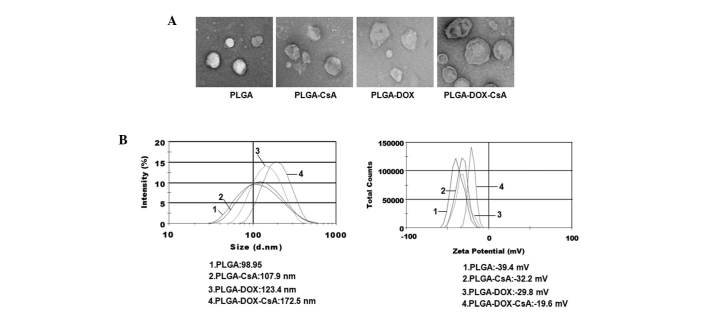
Characteristics of nanoparticles PLGA, PLGA-DOX, PLGA-CsA and PLGA-DOX-CsA prepared as described in the Materials and methods section. (A) the morphology of the nanoparticles was image captured by TEM. (B) Size distribution and (C) ζ potential of nanoparticles were measured using dynamic light scattering analysis. PLGA, poly(D,L-lactide-*co*-glycolide); DOX, doxorubicin; CsA, cyclosporin A; PLGA-CsA, CsA-coated PLGA; PLGA-DOX, DOX-loaded PLGA; PLGA-DOX-CsA, DOX and CsA-loaded PLGA; TEM, transmission electron microscopy.

To synthesize PLGA-DOX, PLGA-CsA and PLGA-DOX-CsA, DOX and CsA (alone or together) were encapsulated into PLGA nanoparticles. TEM was first performed to observe the prepared nanoparticles. As shown in Fig. 2A, all nanoparticles were dispersed as individual particles with a well-defined spherical shape and homogeneously distributed diameters of ~120 nm. Size distribution (Fig. 2B) and ζ potential (Fig. 2C) analyses of the nanoparticles revealed that the average size of free PLGA was 98.57±3.6 nm, PLGA-CsA was 107.9±6.9 nm, PLGA-DOX was 123.5±12.3 nm and PLGA-DOX-CsA was 172.9±14.6 nm, and all nanoparticles were negatively charged (PLGA, −39.4±2.6; PLGA-CsA, −32.3±4.7; PLGA-DOX, −34.6±7.1; and PLGA-DOX-CsA, −20±2.9 mV).

### PLGA or PLGA-CsA nanoparticles reduce the efflux of DOX in A549-Taxol cells

**Figure 3:**
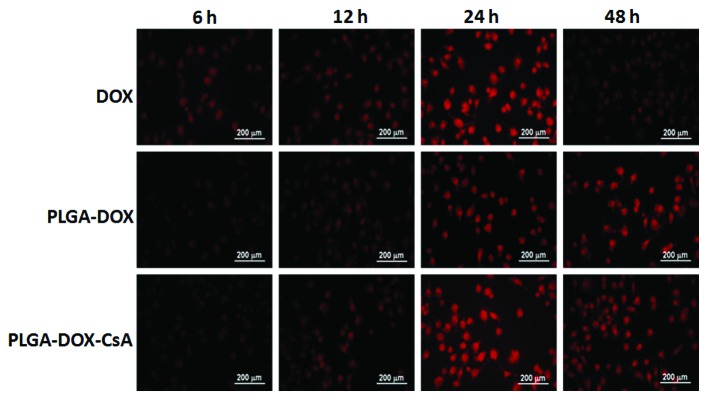
Accumulation of free DOX or nanoparticle-delivered DOX in A549-Taxol cells. DOX (2 μg/ml) that was free or loaded on nanoparticles was incubated with the A549-Taxol cells. Images of the accumulation of DOX in the cells were captured following 6, 12, 24 and 48 h of incubation (Olympus IX71 invert fluorescence microscope; scale bars, 200 μm). DOX, doxorubicin; CsA, cyclosporin A; PLGA-DOX, DOX-loaded PLGA; PLGA-DOX-CsA, DOX and CsA-loaded PLGA.

To investigate whether PLGA or PLGA-CsA increased the accumulation of DOX in the A549-Taxol cells, fluorescence microscopy was used to examine the intensity of DOX. A549-Taxol cells were plated in 12-well plates and incubated with free DOX, PLGA-DOX and PLGA-DOX-CsA, respectively. At various times (6, 12, 24 and 48 h), the cells were washed and images were captured. As indicated in Fig. 3, free DOX was notably reduced at 48 h, while the accumulation of PLGA-DOX, and particularly PLGA-DOX-CsA, remained at a high level.

### PLGA-DOX and PLGA-DOX-CsA effectively inhibit cell viability in vitro

**Figure 4:**
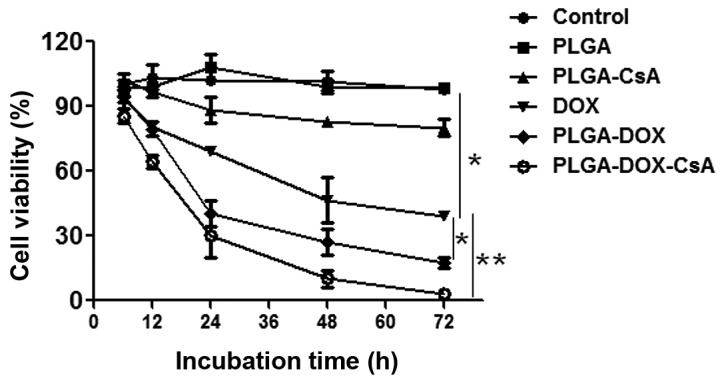
Cell viability following exposure to PBS, PLGA, PLGA-CsA, free DOX, PLGA-DOX and PLGA-DOX-CsA at various culture times at 37 °C. Compared with the free DOX, PLGA-DOX and PLGA-DOX-CsA significantly inhibited cell viability. ^*^P<0.05, vs. PLGA-DOX; ^**^P<0.01, vs. PLGA-DOX-CsA. DOX, doxorubicin; PLGA, poly(D,L-lactide-co-glycolide); CsA, cyclosporin A; PLGA-CsA, CsA-coated PLGA; PLGA-DOX, DOX-loaded PLGA; PLGA-DOX-CsA, CsA-loaded PLGA.

Nanoparticles without DOX (PLGA) were found to have no toxic effects on the cells, as shown in Fig. 4. The free DOX group exhibited proliferation of the A549-Taxol cells compared with the control, PLGA and PLGA-CsA groups (P<0.01). PLGA-DOX and PLGA-DOX-CsA further enhanced the inhibitory function of DOX *in vitro* (P<0.05 and P<0.01, respectively). Furthermore, the CsA-loaded PLGA group reduced the proliferation of A549-Taxol cells compared with the control and PLGA groups (P<0.05).

### PLGA-DOX and PLGA-DOX-CsA enhance antitumor activity

**Figure 5:**
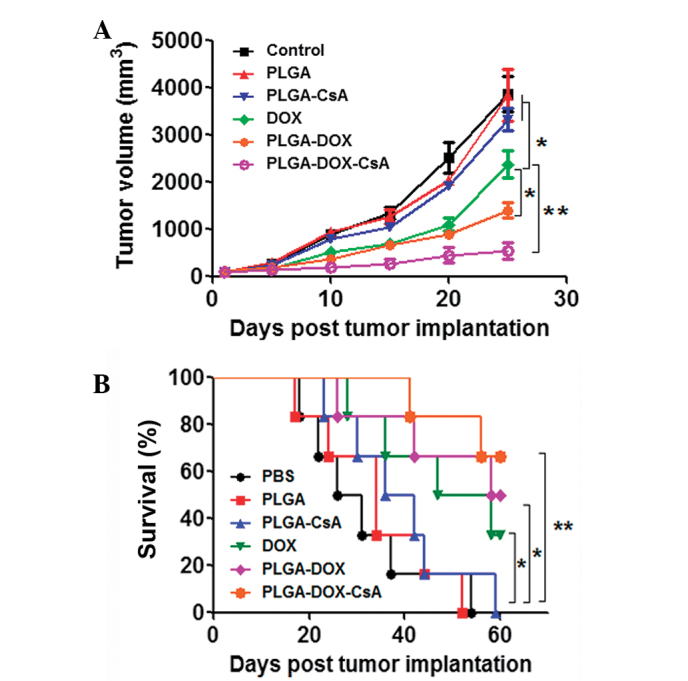
*In vivo* tumor growth and survival rate of tumor-bearing mice. (A) The tumor inhibitory effect of PLGA-DOX and PLGA-DOX-CsA was compared with free DOX, PLGA vector and PBS control in the A549-Taxol tumor model (n=6). DOX and PLGA-DOX-CsA demonstrated significant tumor inhibition. (B) Compared with the PBS, PLGA and PLGA-CsA groups the survival rate of A549-Taxol-bearing mice was significantly improved in the PLGA-DOX and PLGA-DOX-CsA groups. ^*^P<0.05, vs. PLGA-DOX; ^**^P<0.01, vs. PLGA-DOX-CsA. DOX, doxorubicin; PLGA, poly(D,L-lactide-*co*-glycolide); CsA, cyclosporin A; PLGA-CsA, CsA-coated PLGA; PLGA-DOX, DOX-loaded PLGA; PLGA-DOX-CsA, CsA-loaded PLGA.

The *in vivo* antitumor activity of free DOX, PLGA-DOX, PLGA-CsA and PLGA-DOX-CsA was evaluated with A549-Taxol tumor-bearing mice (6 per group). Treatments were performed as i.v. injections of PBS, free PLGA, free DOX, PLGA-DOX, PLGA-CsA or PLGA-DOX-CsA in tumor-bearing mice with average tumor volumes of 100 mm^3^, and this day was designated as day 1. Fig. 5A shows the variations in tumor volume compared with the number of days after treatment in the A549-Taxol tumor-bearing mice. Mice treated with free DOX exhibited effectively inhibited tumor growth compared with the PBS, PLGA and PLGA-CsA groups (P<0.05). As predicted, the PLGA-DOX and PLGA-DOX-CsA groups exhibited improved tumor inhibitory effects compared with the free DOX group (P<0.05 and P<0.01, respectively). The survival rates in Fig. 5B indicated that the co-delivery of DOX and CsA further increased the survival rate of A549-Taxol-bearing mice (P<0.01, vs. PBS, PLGA and PLGA-CsA groups).

## Discussion

Cancer is one of the most significant causes of mortality in humans, and the incidence and mortality rates of cancer are continuously rising (19). A thorough ‘cure for cancer’ remains elusive for a number of reasons. One of the critical reasons is the strong toxic side-effects of free drugs or traditional drug delivery vectors, mainly due to drug leakage prior to reaching the cancer site. In addition, the intrinsic or acquired MDR of cancer is primarily responsible for the final failure of cancer chemotherapy, with >90% of patients with malignant tumors succumbing due to a certain degree of MDR. Therefore, MDR in cancer has become a major obstacle in the chemotherapeutic treatment of numerous types of human cancer (20). Overcoming the currently untreatable MDR in cancer remains important in antitumor research.

Current strategies to overcome tumor MDR generally resort to multi-drug combined chemosensitization, reconstruction of primary drugs and bio-/nanotechnologies. The combined use of two or several strategies is being recognized as a realistic route to successful chemotherapeutic treatment. By integrating multi-drug chemosensitization with nanotechnology, specific nano drug delivery systems based on organic or inorganic nanocarriers have been designed. These systems overcome MDR and also enhance drug efficacy against drug-sensitive and -resistant cancer cells, mainly by improving drug bioaccessibility and chemosensitivity. Varying types of combinations against MDR in cancer have been identified, including the combination of proapoptotic compounds with chemotherapeutics (21), MDR-targeted siRNA with chemotherapeutics (22), nanoparticles co-encapsulating hydrophobic and hydrophilic drugs (23), nanoparticles with precise ratiometric drug loading (24) and efflux pump inhibitors with chemotherapeutics (25).

According to previous studies on drug resistant tumor therapy, PLGA, which contains a solid, polymer-filled core that is more suited for water-insoluble drug payloads as a delivery tool, and CsA (26), which functions as a drug efflux pump inhibitor to further enhance the accumulation and reduce the efflux of DOX, were selected for use in the present study. Following the synthesis and characterization of PLGA-DOX, PLGA-CsA and PLGA-DOX-CsA, the accumulation of free DOX, PLGA-DOX and PLGA-DOX-CsA were initially investigated in A549-Taxol cells and the results demonstrated that the PLGA-DOX and PLGA-DOX-CsA groups exhibited increased accumulation of DOX compared with the naked DOX group. Furthermore, >80% of the A549 cells at 48 h and 90% at 72 h were killed by PLGA-DOX-CsA and ~70% of the cells at 48 h and 80% at 72 h were extirpated by PLGA-DOX at a DOX concentration of 10 mg/ml, while only 40% of the cells were eliminated by free DOX. *The in vivo* antitumor model also indicated that PLGA-DOX and PLGA-DOX-CsA not only inhibited the tumor growth, but also increased the survival rate of A549-Taxol-bearing mice.

Collectively, PLGA is considered to be a safe delivery system tool due to its biodegradability and biocompatibility, since a number of PLGA-based drug systems have been approved by the FDA. This modified delivery vector is further empowered by co-loading with tumor-targeted molecules, tumor-sensitive drugs, inhibitors associated with tumor progression and modulators of drug resistance. In addition, this type of multi-targeted therapeutic method should be a more effective tumor therapeutic method.
